# Oligonucleotide conjugated antibodies permit highly multiplexed immunofluorescence for future use in clinical histopathology

**DOI:** 10.1117/1.JBO.25.5.056004

**Published:** 2020-05-22

**Authors:** Nathan P. McMahon, Jocelyn A. Jones, Sunjong Kwon, Koei Chin, Michel A. Nederlof, Joe W. Gray, Summer L. Gibbs

**Affiliations:** a Oregon Health and Science University, Biomedical Engineering Department, Portland, Oregon, United States; b Quantitative Imaging, LLC, Pittsburgh, Pennsylvania, United States; c Oregon Health and Science University, Knight Cancer Institute, Portland, Oregon, United States; d Oregon Health and Science University, OHSU Center for Spatial Systems Biomedicine, Portland, Oregon, United States

**Keywords:** cyclic immunostaining, oligonucleotide conjugated antibody, multiplexed immunostaining, cancer heterogeneity

## Abstract

**Significance:** Advanced genetic characterization has informed cancer heterogeneity and the challenge it poses to effective therapy; however, current methods lack spatial context, which is vital to successful cancer therapy. Conventional immunolabeling, commonplace in the clinic, can provide spatial context to protein expression. However, these techniques are spectrally limited, resulting in inadequate capacity to resolve the heterogenous cell subpopulations within a tumor.

**Aim:** We developed and optimized oligonucleotide conjugated antibodies (Ab-oligo) to facilitate cyclic immunofluorescence (cyCIF), resulting in high-dimensional immunostaining.

**Approach:** We employed a site-specific conjugation strategy to label antibodies with unique oligonucleotide sequences, which were hybridized *in situ* with their complementary oligonucleotide sequence tagged with a conventional fluorophore. Antibody concentration, imaging strand concentration, and configuration as well as signal removal strategies were optimized to generate maximal staining intensity using our Ab-oligo cyCIF strategy.

**Results:** We successfully generated 14 Ab-oligo conjugates and validated their antigen specificity, which was maintained in single color staining studies. With the validated antibodies, we generated up to 14-color imaging data sets of human breast cancer tissues.

**Conclusions:** Herein, we demonstrated the utility of Ab-oligo cyCIF as a platform for highly multiplexed imaging, its utility to measure tumor heterogeneity, and its potential for future use in clinical histopathology.

## 1 Introduction

Our understanding of cancer has evolved from a view of a collection of cells exhibiting unchecked proliferation to the realization that cancers include heterogenous genetic cell populations able to evade death through oncogenic dysregulation^1^^,^^2^ and complex interactions with the tumor microenvironment.^3^^–^^5^ We owe much of our recent understanding to large cancer genome sequencing efforts that uncovered novel cancer genes and genetic diversity using molecular characterization technologies such as next generation sequencing and quantitative polymerase chain reaction sequencing. However, the translation of complex genomic analyses to gold standard pathological diagnoses remains challenging as conventional immunohistochemical (IHC) and immunofluorescence (IF) staining are limited to approximately two to five antigens per sample.^6^ Additionally, while genomic analyses are a powerful therapeutic tool, they are deployed at the cost of spatial context of biomarker distribution. Recent discoveries have revealed the significance of the spatial relationships between cancer, immune, and microenvironmental cells in response and resistance to therapy,^7^^,^^8^ highlighting the importance of their preservation. Thus, to further understand the diagnostic and prognostic implications of these relationships, a molecular profiling technology to measure both expression and spatial context of biomarkers, while preserving both, is required.

While conventional immunolabeling techniques permit simultaneous expression and spatial context analysis of proteins, their spectral limit (i.e., approximately two to five antigens per sample) requires a modified solution to understand the complexity of cancer proteomics *in situ*. To overcome this limitation, a number of strategies have been developed to increase single sample specific-antigen labeling, but to date, none of these methods can be seamlessly integrated into routine clinical histopathology. Mass spectroscopy techniques, such as multiplexed ion beam imaging (MIBI)^9^^–^^11^ and cytometry by time of flight (CyTOF),^12^ offer significant multiplexing capabilities using rare earth metal mass tags with simultaneous detection of up to 40 antigens in formalin fixed paraffin embedded (FFPE) tissues demonstrated.^10^ However, integration into clinical histopathology will present challenges, including (1) nontrivial generation of isotope labeled probes, (2) detection of low abundance antigens, which is generally poor, and (3) difficulty in translation to the routine pathology lab due to instrumentation expense and required technological expertise. IF permits higher dimensionality immunostaining than IHC, but the maximum number of fluorophores that can be visualized on a single sample using conventional fluorescence microscopy is five and using unmixing techniques it is improved to seven.^6^^,^^13^^,^^14^ Various techniques using sequential cycles of fluorescent tagging, imaging, and bleaching^15^^–^^17^ or dissociation of affinity tags^6^^,^^18^^,^^19^ have demonstrated multiplexing capabilities of up to 61 immunostained targets in a single sample.^15^ Cyclic IHC techniques that facilitate staining of up to 12 antigens on a single sample stained and imaged one-by-one have also been developed.^20^ Although these techniques improve multiplexing capabilities, they will be difficult to incorporate into clinical histopathology because (1) antigenicity of the tissue is affected by the bleaching and destaining methods^21^ and (2) steric hindrance issues can occur as cycle number increases.^9^^,^^22^ Antibody barcoding techniques [e.g., Nanostrings,^23^^,^^24^ CODEX,^25^ DNA exchange imaging (DEI),^26^ immuno-SABER,^27^ etc.) have also been developed, demonstrating the ability for highly multiplexed cyclic fluorescence imaging using nondestructive signal removal techniques. The Nanostrings technology has been shown to visualize up to 90 antigens in a single cellular sample,^23^ and after extension to tissue, has demonstrated visualization of up to 32 antigens; however, this technology is not yet feasible for whole tissue imaging.^24^ Nanostrings assessment of entire tissue sections for clinical histopathology will be time consuming and costly as spot scanning at $100\times 100\;\mu m^{2}$ resolution for detection is required, and since barcodes are released from tissue for detection, subcellular resolution is not possible. In contrast, CODEX, DEI, and immuno-SABER have demonstrated the capability of subcellular resolution paired with high-dimensional imaging. However, due to protocol complexity, instrumentation expense, and the necessary technological expertise, they cannot be readily integrated into the clinical workflow. Therefore, although multiplexed protein detection is possible, none of the current methods will readily translate to routine clinical histopathology.

Our novel, highly multiplexable cyclic IF (cyCIF) technique is capable of generating multiparametric images for quantifying biomarker expression and distribution and is readily translatable to the clinical setting. While similar to other antibody barcoding techniques, the workflow described herein has minimal variation from indirect IF tissue staining procedures that are commonplace in the clinical laboratory setting. Termed antibody conjugated oligonucleotide (Ab-oligo) cyCIF, it preserves tissue antigenicity and lends itself to ready integration into clinical workflows. Similar to the Nanostrings technology, our Ab-oligo cyCIF exploits *in situ* hybridization of complementary oligonucleotides for biomarker labeling and the oligo modifications to facilitate signal removal for sequential rounds of fluorescent tagging and imaging. In our technology, a single-stranded oligo [docking strand (DS)] is conjugated to the primary antibody. Subsequent introduction of a complementary single-stranded oligo [imaging strand (IS)] conjugated to a conventional Alexa Fluor (AF) fluorophore (e.g., AF488, AF546, and AF647) facilitates specific on tissue fluorescent labeling through *in situ* hybridization and imaging with any conventional fluorescence microscope. The presence of a photocleavable linker (PCL) between the fluorophore and oligo sequence facilitates signal removal to levels of autofluorescence after ultraviolet (UV) light exposure and prior to subsequent staining cycles, while maintaining hybridization between the DS/IS pair after imaging to diminish the possibility for any cross talk between staining cycles. The advantages of our method over other multistaining methods include (1) all Ab-oligos are applied in a single mixed cocktail at the beginning of the study, preventing steric hindrance, and (2) application of all Ab-oligos in a single staining step drastically reduces overall staining time as only a single long antibody incubation step is required. Ab-oligo cyCIF is therefore able to visualize endogenous protein expression while maintaining spatial context *in situ*, which is critical to identifying heterogenous cell populations within tumors. Using our Ab-oligo cyCIF platform, we have generated up to 14 color images on multiple breast cancer FFPE samples as proof-of-concept of our cyCIF technology.

Quantification of protein signatures *in situ* could revolutionize cancer care in much the same way that genomic analyses have changed the landscape of cancer diagnosis and therapy selection. Reliable and robust quantitative analysis of protein signatures will require a consistent, multiplexed, *in situ* staining method with a coupled computational visualization and analysis platform that generates repeatable biomarker and clustering signatures. Additionally, to influence clinical decisions, this methodology must integrate seamlessly into the clinical histopathology workflow. Herein, we validate our Ab-oligo cyCIF methodology, demonstrating its capability to produce high-dimensionality data from a single tissue sample with the potential to unravel the complexity of a tumor with the capability for ready translation into the clinical setting.

## 2 Materials and Methods

### 2.1 Primary Antibody Conjugation

Monoclonal antibodies were purchased from AbCam (Cambridge, UK), Thermo Fisher Scientific (Waltham, Massachusetts), Biolegend (San Diego, California), or Cell Signaling Technology (Danvers, Massachusetts) to the following targets: cytokeratin 5 (CK5), cytokeratin 8 (CK8), cytokeratin 19 (CK19), proliferating cell nuclear antigen (PCNA), Ki-67, E-cadherin (E-Cad), human epidermal growth factor receptor 2 (HER2), α-smooth muscle antigen (α-SMA), CD44, cytochrome c oxidase (CoxIV), CD4, and CD3 (Table 1). A unique dibenzocyclooctyne (DBCO) or amine-terminated single-stranded oligonucleotide (DS, 28 mer in length) was obtained from Integrated DNA Technologies (Coralville, Iowa) to conjugate to each primary antibody. Prior to modification, the immunoglobin G (IgG) antibodies were purified from storage buffers, including an azide reagent using 50 kDa Amicon filters (EMD Millipore, Burlington, Massachusetts). Antibody modification and oligonucleotide (oligo) conjugation were performed using either the SiteClick™ antibody azido modification kit (Thermo Fisher Scientific) or the Solulink™ antibody modification kit (TriLink Biotechnologies, San Diego, California) following the manufacturer’s instructions, explained briefly as follows. The SiteClick kit cleaved the carbohydrate domains on the heavy chains of the monoclonal IgG antibodies using β-galactosidase, enabling the addition of an azido moiety. The DBCO-terminated DS was mixed with the azido-modified whole IgG and standard copper-free click chemistry reagents, resulting in site-specific labeling of the antibodies with their unique oligo sequence (Ab-oligo). Using the Solulink kit, the antibody was modified at the available lysine residues, where a S-HyNic (part# S-9011-1-01, TriLink Biotechnologies) small molecule was conjugated. Simultaneously, the amine-terminated DS was covalently modified to contain a 4-FB (part# S-9011-1-02, TriLink Biotechnologies) small molecule on the $5^{\prime }$ end. The 4-FB modified DS and S-HyNic conjugated antibody were mixed together at a molecular target ratio of 20 oligos to 1 antibody and the N-hydroxysuccinimide (NHS) ester reaction occurred in the presence of the Turbolink™ catalyst (part# S-9011-1-05, TriLink Biotechnologies). Excess oligo was removed from the Ab-oligo conjugates using molecular weight cut-off spin columns. After purification, the absorbance of the Ab-oligo conjugate was measured on a Spectramax M5 (Molecular Devices, San Jose, California). The maximum absorbance of the antibody and conjugated DS was measured at 280 and 260 nm, respectively. Using the estimated extinction coefficient of $210,000\;M^{-1}{\mathrm{cm}}^{-1}$ for all antibodies and the manufacturer reported extinction coefficient for each DS, the approximate Ab-oligo conjugation ratios (CR) were calculated to quantify the average number of oligos bound to an antibody.

**Table 1:** Oligonucleotide conjugated antibodies (Ab-oligos).

	Biomarker	Vendor	Ab-oligo CR	FL CR	Stained tissue	DS oligonucleotide sequence
Cell state	CK5	AbCam	1.20	0.52	Normal breast	5′-AATATGGAATTCGTCCGAGCCCGTCAAG-3′
	CK8	AbCam	1.27	0.58	Normal breast	5′-CCCAAAGGTACCCGTCGTAGTAACAAAG-3′
	CK19	Biolegend	0.85	1.74	Normal breast	5′-AATAATGTCGACTACGCCTGACCGTCGT-3′
	PCNA	AbCam	1.24	0.95	MDA-MB-468	5′-ATTTACGGTACCAGTCGTACAAGACGGG-3′
	Ki-67	AbCam	1.15	0.74	MDA-MB-468	5′-AAGAAGCTGCAGTGCGATTTAAGGTCGG-3′
	E-Cad	AbCam	1.13	0.48	Normal breast	5′-TTATATCCATGGCCGCAATCGCCTCGAT-3′
Breast cancer	HER2	Themo	1.09	1.72	Sk-Br-3	5′-AAAATTCGGCCGTGAAGTCGTTCGCCAG-3′
Stroma	α-SMA	AbCam	1.24	0.62	Normal breast	5′-ATATATGGATCCCTGGCGTGGTTCGTCG-3′
	CD44	AbCam	1.30	0.70	MDA-MB-468	5′-AATATAGTCGACCGCCGATGCTTCGTGG-3′
Architecture	CoxIV	CST	1.26	1.38	Sk-Br-3	5′-TTAATTCGGCCGCGCTTACGGACTCAGT-3′
Immune	CD4	AbCam	1.20	0.88	Tonsil	5′-ATCTTTCAGCTGCGAACGTAAACCTCGG-3′
	CD3	AbCam	1.48	0.91	Tonsil	5′-TACACCAAGCTTTAAACTGCGAGCGACA-3′

Note: CR, Ab-oligo conjugation ratio; FL CR, antibody to fluorophore conjugation ratio for direct IF comparison with Ab-oligo cyCIF.

An aliquot of the same primary antibody used for oligo conjugation was directly conjugated to AF555 NHS ester (AF555 NHS, Thermo Fisher Scientific). The starting antibody concentration was determined by measuring the absorbance at 280 nm prior to any conjugation chemistry. The primary antibody was again purified of any storage buffers (e.g., sodium azide) and buffer exchanged into 1× PBS, pH 8.3 with a 10 kDa Amicon filter. AF555 NHS was resuspended at 10 mM in anhydrous dimethyl sulfoxide and added to the antibody at a ratio of 10 fluorophore molecules to 1 antibody in a reaction volume of 1 mL. The mixture was rocked gently at 4°C for 3 h protected from light. The antibody conjugate was then purified with a 10 kDa Amicon filter. Absorbance was measured at 280 and 555 nm to calculate the antibody and fluorophore concentrations, respectively, enabling quantification of the CR (FL CR) or the average number of fluorophores bound to an antibody.

### 2.2 Formalin Fixed Paraffin Embedded Tissue and Cell Samples for Antibody Validation

Deidentified human FFPE tissue blocks were obtained from the Oregon Health and Science University Knight Biolibrary. The functionality of oligonucleotide and fluorophore conjugated antibodies was validated on FFPE blocks of normal breast tissue, normal tonsil tissue, and FFPE cell buttons generated from MDA-MB-468 or Sk-Br-3 breast cancer cell lines. All cell lines were originally purchased from ATCC (Old Town Manassas, Virginia) and maintained at less than 25 passages for all experiments. The tissue or cell button type known to express the target of interest was used for validation of each primary antibody (Table 1). Five micrometer sections of the selected FFPE block were captured onto 1×3  in. Superfrost™ plus slides (Thermo Fisher Scientific), which were baked at 65°C in a hybridization oven for times ranging from 30 min to overnight prior to commencing staining.

### 2.3 Antibody Staining on FFPE Samples

The baked FFPE slides were deparaffinized at room temperature (RT) in xylenes (2×10  min⁡ washes). The tissue was then gradually rehydrated in ethanol and water solutions as follows: 100% ethanol (2×10  min⁡), 95% ethanol (5 min), 70% ethanol (5 min), and then, 50% ethanol (5 min). The tissue was then rinsed in 100% deionized water (${\mathrm{diH}}_{2}O$) followed by a wash in 1× PBS, pH 7.4 for 10 min. A two-step antigen retrieval procedure was performed in a Pascal Pressure Cooker (Dako, Santa Clara, California). RT solutions of 10 mM sodium citrate, pH 6, 1× tris hydrochloride (HCl), pH 8, and ${\mathrm{diH}}_{2}O$ were placed in the pressure cooker in three individual plastic buckets immersed in 500 mL of ${\mathrm{diH}}_{2}O$, which covered the bottom of the chamber. The slides were immersed in the sodium citrate buffer and the pressure cooker lid was secured. The temperature was increased to 125°C over a 15-min period, where the pressure reached ∼15  psi. The pressure cooker was removed from the heat, and the temperature and pressure were allowed to decrease to 90°C and 0 psi, respectively, over ∼25  min. The residual pressure was released as the lid was removed and the slides were rinsed in the hot ${\mathrm{diH}}_{2}O$. The slides were immediately placed in the hot tris-HCl buffer, where they were incubated for 10 min with the lid on the pressure cooker. The slides were then transferred back to the hot ${\mathrm{diH}}_{2}O$ and brought back to RT by slow addition of RT water to the vessel. After 5 min in RT ${\mathrm{diH}}_{2}O$, the slides were washed in PBS, pH 7.4 at RT for an additional 5 min.

Antibody staining studies were completed using four groups for staining pattern validation, including (1) conventional indirect IF with unconjugated primary and fluorophore conjugated secondary antibody, (2) the Ab-oligo conjugate plus the same fluorophore conjugated secondary antibody, (3) the Ab-oligo conjugate plus the complementary fluorophore conjugated IS, and (4) the fluorophore labeled primary. The same overall staining procedure was used for each group, explained as follows. The antigen retrieved slides were blocked at RT for 30 min in an Ab-oligo blocking and dilution buffer, which contained 2% bovine serum albumin (BSA, bioWORLD, Dublin, Ohio), 0.5  mg/mL sheared salmon sperm DNA (Thermo Fisher Scientific), and 0.5% dextran sulfate (Sigma-Aldrich, St. Louis, Missouri) in 1× PBS, pH 7.4. The Ab-oligo conjugate was diluted in the Ab-oligo blocking and dilution buffer to a final protein concentration of 15  μg/mL. The tissue sections were covered with 40 to 100  μL of the diluted Ab-oligo conjugate and incubated at 4°C overnight in a humidified chamber. The next day, the sections were washed with a 2× saline-sodium citrate (SSC) buffer, pH 7 (VWR, Radnor, Pennsylvania) for 15 min. The sections were fixed in 2% paraformaldehyde (PFA, Sigma-Aldrich) for 15 min at RT and then washed again in a 2× SSC buffer (3×5 min⁡).

For groups (1) and (2), AF555 conjugated secondary antibody was added at 350 nM. For group (3), complementary IS labeled with AF546, diluted to 350 nM in an IS dilution buffer containing 2% BSA, 0.5  mg/mL sheared salmon sperm DNA, and 0.5% dextran sulfate in a 2× SSC buffer was added. The sections were incubated with IS at RT protected from light for 45 min. The IS was removed, and the sections were washed in a 2× SSC buffer (3×5 min⁡). The slides were protected from light for the remainder of the staining procedure. Group (4) did not require any secondary detection reagent. 4',6-diamidino-2-phenylindole (DAPI, Thermo Fisher Scientific) was applied to all stained sections at 300 nM for 10 min at RT, and the slides were then washed in a 2× SSC buffer (2×5 min⁡). The stained slides were mounted in Fluoromount-G (Southern Biotech, Birmingham, Albama) and coverslipped for imaging.

### 2.4 Fluorescence Microscopy, Image Visualization, and Image Analysis

Antibody stained slides were imaged on a Zeiss AxioImager.M2 with motorized XY scanning stage (Carl Zeiss AG, Oberkochen, Germany) equipped with a CoolSNAP HQ2 14-bit CCD camera (Photometrics, Tucson, Arizona). Fluorescence images were collected using three channels for IS visualization. Depending on the fluorophore used for IS labeling, the following Zeiss filter sets were used: 38HE (Cy2/AF488), 43HE (Cy3/AF555), and 50 (Cy5/AF647). Excitation light was filtered using the following bandpass (BP) filters 470/40 (HE), 550/25 (HE), and 640/30, for AF488, AF546/AF555, and AF647, respectively. Emission light was filtered using the following BP filters 525/50 (HE), 605/70 (HE), and 690/50, for AF488, AF546/AF555, and AF647, respectively. DAPI was imaged using Zeiss filter set 49 (UV/DAPI), where excitation was filtered at 365 nm and emission was filtered using a 445/50 BP filter. Single-color, single field-of-view images were collected using an X-Cite 120Q (Excelitas Technologies Corporation, Waltham, Massachusetts) light source at 40× (Plan-Apochromat, 0.95NA) magnification. Images of the entire FFPE section were collected with 10% overlap at 20× (Plan-Apochromat, 0.8NA) magnification, where overlapping images were tiled into a single tissue map. Each fluorescence channel was registered using QiTissue software (Quantitative Imaging Systems, LLC, Pittsburgh, Pennsylvania).

Signal-to-background ratio (SBR) was calculated from collected images using a Python script to extract mean fluorescence intensity of marker-specific fluorescent signal and separate the background from each image (DOI: 10.5281/zenodo.3738745). To quantify the marker specific signal, a threshold was established to create a binary mask to extract mean fluorescent intensity from pixels only in positively stained regions of the image. The threshold was established using ImageJ v1.51 (NIH), where the histogram minimum and maximum were adjusted to only display positive pixels. Positive pixels were displayed white and equal to one in the image array while negative pixels were black and equal to zero. For example, a binary mask for a membrane specific marker in which only tissue membrane pixels were white would be created. The binary image array of ones and zeros was used to filter pixels to be counted if their binary image array location had a value of one. To measure background fluorescence, the binary mask was inverted to measure fluorescent signal from pixels not in positively stained regions of the tissue, where, now, the background pixels previously valued as zero in the binary array were equal to one and included in the background fluorescence quantification. SBRs were calculated for image data sets by dividing the signal fluorescent intensity by the background fluorescent signal intensity. Mean fluorescence intensity was used in lieu of SBR for data reporting signal removal in which signal is only present prior to signal removal, making SBR an inappropriate metric for image analysis after signal removal.

### 2.5 Ab-oligo Conjugate Titration

The previously detailed FFPE antibody staining procedure was used to stain normal breast tissue with the CK8 Ab-oligo conjugate after being diluted in an Ab-oligo dilution buffer to final concentrations of 15, 5, 1.5, and 0.15  μg/mL. Images were collected at 60 ms with varying contrast settings to display positive staining at all Ab-oligo concentrations. The images were then used to calculate mean intensity of CK8 specific positive staining, as previously described, to evaluate the optimal staining concentration.

### 2.6 Ab-oligo IS Titration

The previously detailed FFPE antibody staining procedure was used to stain normal breast tissue with E-Cad or α-SMA Ab-oligo conjugates and triple negative breast cancer tissue with the Ki67 Ab-oligo conjugate. The complementary IS was diluted in an IS dilution buffer and added to the Ab-oligo stained slides at 100, 250, 350, 500, 750, or 1000 nM. Images were collected at 300 ms for E-Cad, 10 ms for α-SMA, and 500 ms for Ki67 Ab-oligo conjugates. The displayed images have varying contrast settings to exhibit positive staining pattern at all IS concentrations. The images were used to calculate SBR, as previously described, which was used to evaluate the optimal staining concentration.

### 2.7 Optimal IS Fluorophore Configuration

Sk-Br-3 cell buttons were stained with the HER2 Ab-oligo conjugate, and normal breast tissue was stained with the E-Cad Ab-oligo conjugate using the previously described FFPE staining protocol. IS with fluorophore labeled on either the $5^{\prime }$ (i.e., one fluorophore) or both the $5^{\prime }$ and $3^{\prime }$ ends (i.e., two fluorophores) was used for detection. Ab-oligo stained cell buttons or tissues were subsequently stained with 250 nM of the one fluorophore or two fluorophores labeled IS. Images of each stained tissue were collected at 3000 ms for HER2 and 175 ms for E-Cad and were used to calculate SBR to determine the optimal IS configuration.

### 2.8 Signal Removal Using Photocleavable Linkers with Varied IS Lengths

Normal breast tissue was stained with the E-Cad Ab-oligo conjugate using the previously described FFPE staining protocol. ISs of varied lengths (28, 27, and 26 mer) that contained a PCL and AF546 fluorophore on both the $3^{\prime }$ and $5^{\prime }$ ends were used for staining at 350 nM. As a positive control, a 28 mer IS with AF546 on both the $3^{\prime }$ and $5^{\prime }$ ends without PCLs was used. Images of the stained tissues were collected at 600 ms. The mean fluorescence intensity of each image was calculated to determine the optimal IS length. Following imaging, the slides were treated with UV light for 15 min on a UVGL-55 Handheld UV lamp (UVP, Upland, California) through the cover glass. The UV-treated slides were placed vertically in 0.1× SSC for 5 min, allowing for removal of the cover glass. The slides were then washed 10 times with 0.1× SSC and mounted using Fluoromount-G prior to imaging using the same settings. The mean fluorescence intensity of the image was again quantified to determine the amount of retained signal.

### 2.9 Multiplexed Ab-oligo Staining and Imaging

HER2+ BC and BC tissue microarray (TMA) FFPE samples were stained with a cocktail of the 14 Ab-oligo conjugates using the previously described staining protocol. The 12 antibodies outlined in Table 1 as well as an oligo conjugated estrogen receptor (ER) and PD-1 antibodies were mixed at a concentration of 15  μg/mL per antibody into a single cocktail for staining. The tissues stained with the Ab-oligo conjugates were labeled with IS in rounds, where IS labeled with distinct fluorophores and complementary to three Ab-oligos were stained at 350 nM in each staining round. Serial sections were used for staining with the Ab-oligo cocktail or IS only as a negative control. Images were collected in all three channels (AF488, AF546, and AF647), followed by a separate image of the DAPI channel. All stained slides were treated with UV light for 15 min followed by washing 10 times with 0.1× SSC and remounted with Fluoromount-G. Finally, the slides were imaged with the same settings used prior to UV treatment to quantify any remaining signal. Subsequent rounds of IS addition, imaging, and signal removal were repeated until all Ab-oligo conjugates were imaged.

### 2.10 Tissue Damage Quantification of Ab-oligo cyCIF

DAPI images from each round of cyclic staining were analyzed using ilastik^28^ v1.3.3 (European Molecular Biology Laboratory) to quantify the number of nuclei present in the first round and quantify any tissue loss in subsequent rounds. First, a pixel classifier was developed in the Pixel Classification pipeline. The classifier was trained and applied on the full tissue image for each tissue sample to stratify pixels as either a pixel of a cell nucleus or as a background pixel. A map in which each pixel was identified as either a nuclear or background pixel was then used in the boundary-based segmentation with multicut pipeline to create objects from the classified pixels. A watershed algorithm was applied to plant watershed seeds inside each nucleus. The boundary of each watershed object was then exported as the object segmentation map. The object segmentation map was put into the object classification pipeline, where a classifier was trained to identify each object as either background or a cell. After applying the classifier to the full tissue image, a comma-separated values table identifying each object as either background or cell was exported. The number of cell objects for each image was then counted to quantify tissue loss per round of cyclic staining.

## 3 Results

### 3.1 Staining Pattern Validation of Ab-oligo Conjugates

**Fig. 1:**
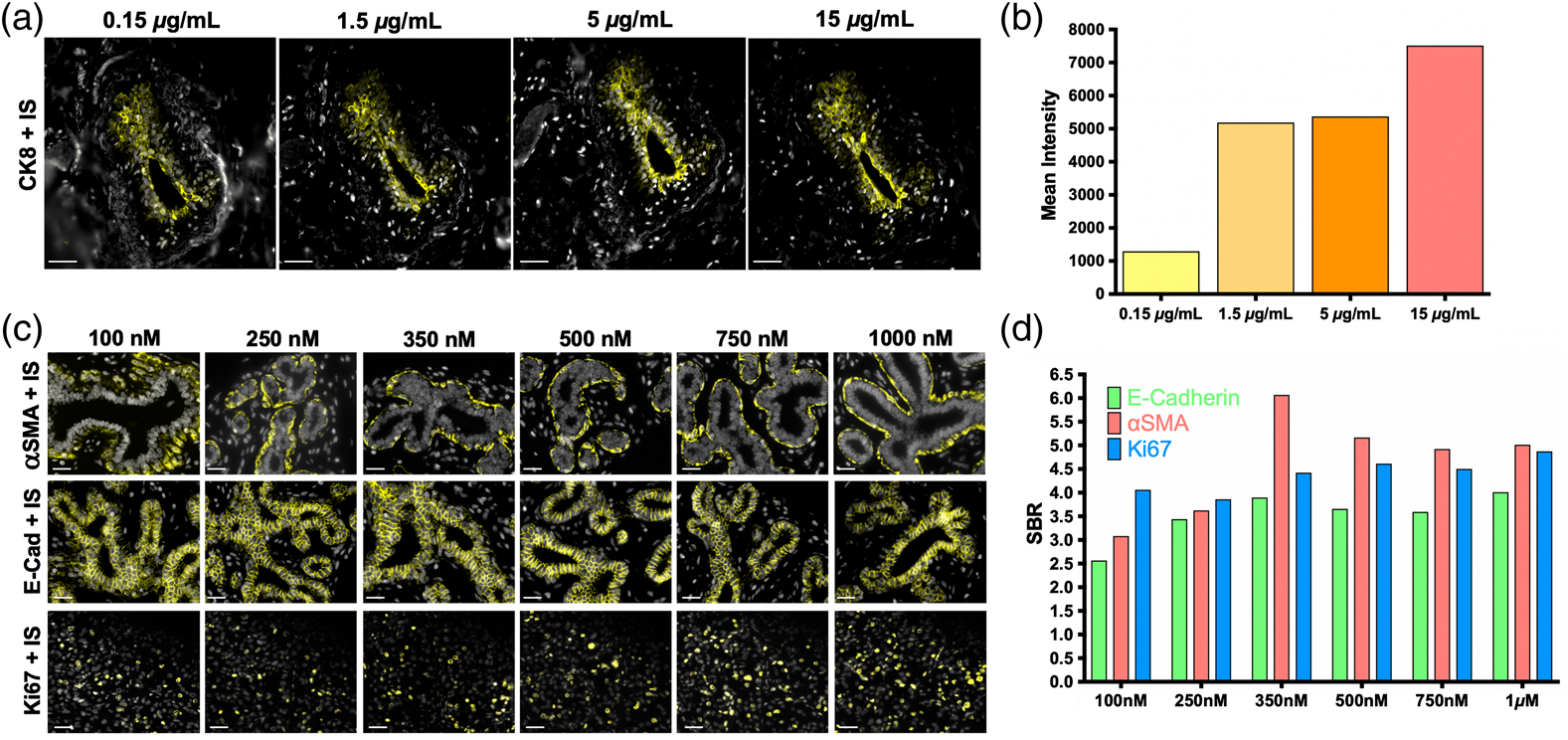
Ab-oligo conjugate and IS titration for optimal staining. (a) The CK8 Ab-oligo conjugate was titrated (0.15 to 15  μg/mL) onto serial sections of normal breast FFPE tissue and equivalent concentrations of IS were applied to all tissue samples for visualization. Images are displayed with contrast and gain optimized for visualization of the positive CK8 staining pattern generated at each antibody conjugate concentration. (b) Image quantification showed the highest mean fluorescence intensity using 15  μg/mL antibody concentration for tissue staining. (c) IS was titrated (100 to 1000 nM) onto FFPE tissue with equivalent Ab-oligo conjugate concentrations present of α-SMA (staining completed on normal breast tissue, top), E-Cad (staining completed on normal breast tissue, middle), and Ki-67 (staining completed on breast cancer tissue, bottom). (d) The SBR was calculated for each concentration tested for all biomarkers. The 40-μm scale bars are displayed in all images.

**Fig. 2:**
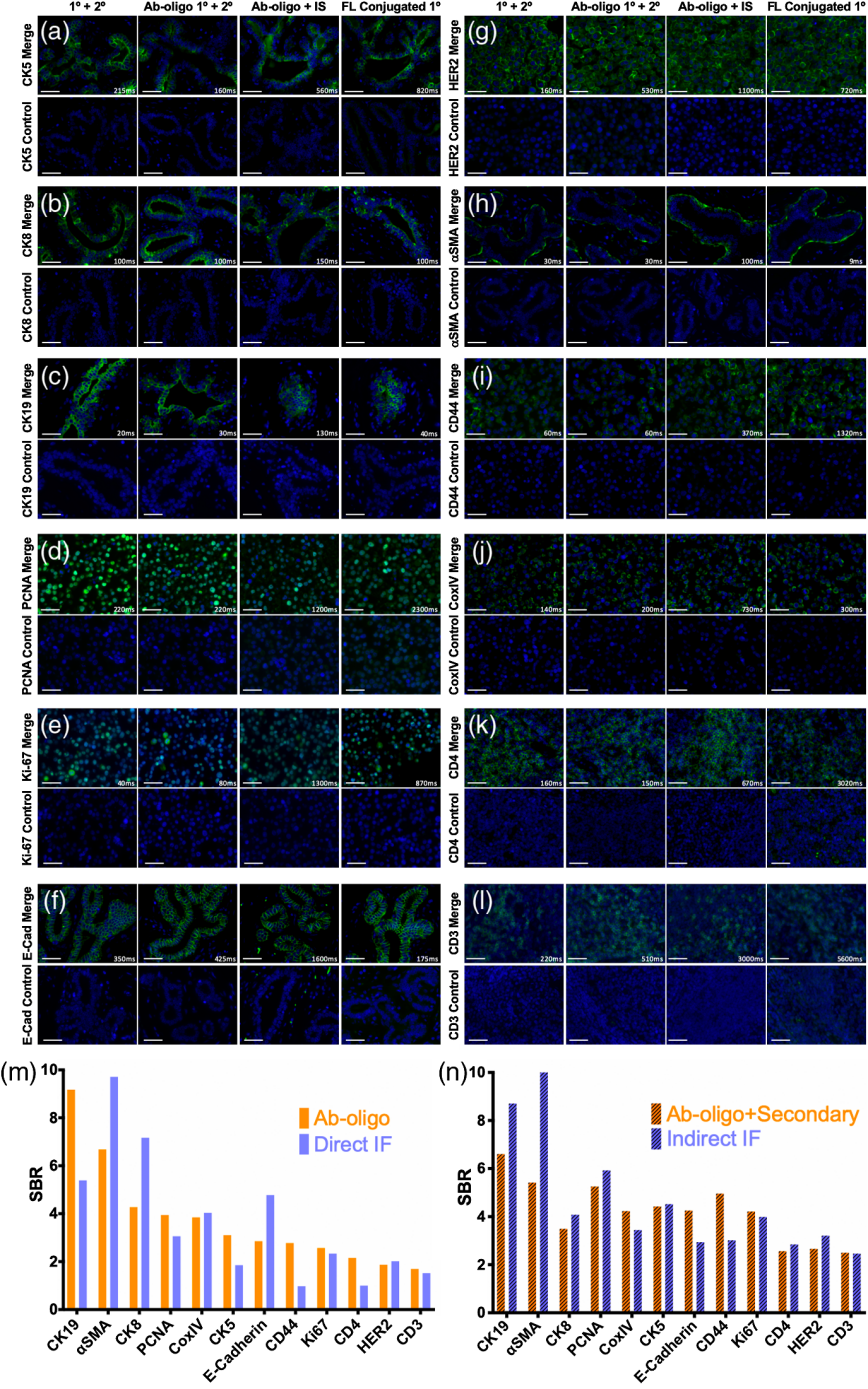
Ab-oligo conjugate staining validation. The staining pattern of each Ab-oligo conjugate was validated by staining serial sections using conventional indirect IF (1°+2°), the Ab-oligo conjugate (1°) detected with a conventional fluorophore-labeled secondary antibody (2°), the Ab-oligo conjugate detected using the complementary IS (Ab-oligo+IS), and direct IF using fluorophore (FL) conjugated 1° antibody. The appropriate negative control image is located below their corresponding antibody stained image. Ab-oligo staining pattern was verified for (a) CK5, (b) CK8, (c) CK19, (d) PCNA, (e) Ki67, (f) E-Cad, (g) HER2, (h) α-SMA, (i) CD44, (j) CoxIV, (k) CD4, and (l) CD3. SBR was calculated for (m) each indirect IF and Ab-oligo+2° image and compared with (n) SBR calculated for each fluorophore (FL) conjugated 1° antibody and Ab-oligo+IS image. The 40-μm scale bars are displayed in all images.

Optimal Ab-oligo conjugate staining concentrations were evaluated on tissue positive for CK8 [Fig. 1(a)], where titration of the Ab-oligo conjugate was performed from 0.15 to 15  μg/mL. Quantification of the CK8-specific fluorescent signal of the same structure in serial FFPE tissue sections showed the highest mean intensity using 15  μg/mL of the Ab-oligo conjugate [Fig. 1(b)]. Subsequently, the optimal IS staining concentration was assessed on tissues positive for α-SMA, E-Cad, and Ki67 [Fig. 1(c)], where IS was titrated from 100 to 1000 nM. The resulting images were qualitatively and quantitatively assessed, showing increased SBR from 100 to 350 nM IS concentrations, with little SBR increase with further increases in IS concentration [Fig. 1(d)], resulting in the selection of 350 nM IS as the optimal staining concentration. A panel of 12 antibodies, selected to elucidate the cell state, breast cancer epithelial cells, stromal compartment, tissue architecture, and immune infiltrate of HER2+ breast cancers (Table 1), were conjugated to a unique DS. The staining pattern of each Ab-oligo conjugate with its complementary IS was confirmed using qualitative comparison to conventional indirect IF staining with an unconjugated primary, indirect IF staining using the Ab-oligo conjugate as the primary antibody, and direct IF staining using a fluorophore-conjugated primary antibody. Negative control tissue was stained with secondary only, IS only, or without any detection reagent (Fig. 2). The staining patterns for all 12 antibodies were qualitatively similar between conventional indirect IF, Ab-oligo indirect IF, Ab-oligo with IS detection, and direct IF staining, demonstrating that oligonucleotide conjugation did not substantially change the affinity of the primary antibody. Four of the 12 antibodies showed similar SBR between conventional indirect IF and Ab-oligo indirect IF staining [Figs. 2(a), 2(e), 2(l), and 2(m)]. Five of the 12 antibodies resulted in a lower SBR when comparing Ab-oligo indirect IF with conventional indirect IF staining [Figs. 2(b), 2(c), 2(d), 2(h), and 2(i)]. The other three Ab-oligo conjugates stained with secondary antibody had higher SBRs than conventional indirect IF staining [Figs. 2(f), 2(j), and 2(k)]. As expected, Ab-oligo with IS staining required longer exposure times than either indirect IF method, generally resulting in decreased SBRs as compared with indirect IF. This required increase in exposure time can be attributed to the signal gain obtained using secondary antibodies that was not possible using the Ab-oligo with our IS staining strategy. As a more direct comparison, primary antibody directly conjugated to AF555 was used to stain serial sections and SBR was quantified for comparison. Overall, similar SBRs were calculated for Ab-oligo detected with IS and direct IF staining; however, there were still sizable differences depending on the stained antigen. For all 12 selected antibodies, the negative control demonstrated that minimal nonspecific background contributed to the overall signal as only nuclear DAPI fluorescence was visible in the normalized control images (Fig. 2). Thus, all 12 Ab-oligo conjugates provided positive staining for the antigen of interest and were able to generate sufficient signal for visualization by conventional fluorescence microscopy.

### 3.2 Optimal IS Design

**Fig. 3:**
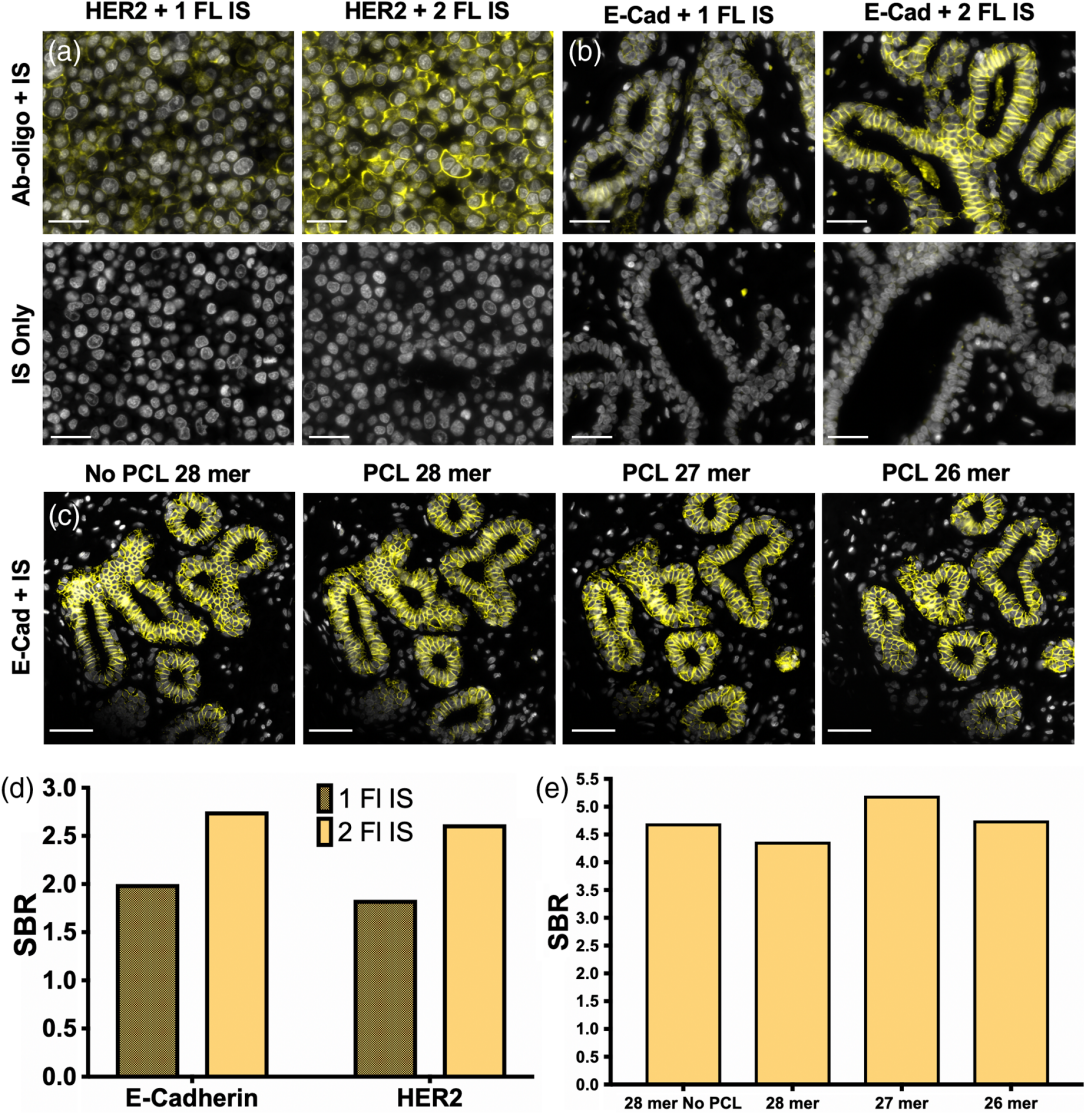
IS design optimization. An IS labeled with a second fluorophore was compared with the original IS design with a single fluorophore for (a) HER2 and (b) E-Cad. (c) IS length with and without a PCL was also investigated using E-Cad labeling, (d) Images were quantified to calculate the generated SBR using an IS labeled with one or two fluorophores as well as (e) with different oligonucleotide lengths (i.e., 26, 27, or 28 nt). The 40-μm scale bars are displayed in all images.

The measured average CR of the 12 Ab-oligo conjugates was near one (average Ab-oligo CR=1.22, Table 1). Thus, a single IS was expected to hybridize to each Ab-oligo conjugate during staining, accounting for the relatively long exposure times compared with indirect or direct IF, where multiple secondary antibodies or fluorophores were available for signal detection, respectively. In an effort to increase the Ab-oligo staining intensity, the IS was modified to contain a fluorophore on both the $3^{\prime }$ and $5^{\prime }$ ends (i.e., two fluorophore design). The Ab-oligo staining intensity was quantitatively compared using identical IS sequences with the one or two fluorophore design for HER2 [Fig. 3(a)] and E-Cad [Fig. 3(b)]. As expected, the additional fluorophore increased the staining intensity, resulting in improved SBR [SBR improvement with two fluorophores versus one fluorophore design: E-Cad=27.4% and HER2=29.9%, Fig. 3(d)]. Due to the SBR increase for both HER2 and E-Cad, the IS design with fluorophore labeled on both the $3^{\prime }$ and $5^{\prime }$ ends (i.e., two fluorophores) was selected for all future studies.

### 3.3 Photocleavable Linkers Enable Complete Signal Removal

PCLs were used as the signal removal strategy, where the size of the PCL and conjugated fluorophore was equivalent to approximately two nucleotides (nt). A study was conducted to determine whether the size of the PCL and fluorophore label created steric hindrance at the $3^{\prime }$ end of the IS binding to the DS using the exact 28 mer IS complement to the antibody conjugated DS. Staining using the E-Cad Ab-oligo with a 28 nt IS sequence without PCL was compared with staining using IS of 28, 27, or 26 nt in length with PCL. Each IS was used to stain a consecutive tissue section as a negative control [Fig. 3(c)]. Quantified mean fluorescence intensity was similar for all IS containing the PCL, regardless of sequence length [Fig. 3(e)]. PCL signal removal was validated after each sample was treated with UV light, resulting in complete signal removal for all PCLs containing IS but maintained staining pattern in the 28 nt IS without PCL [Figs. 3(c) and 3(e)]. Although E-Cad staining intensity was not strongly affected by IS length, the 26 nt IS length was selected for further use to ensure that steric hindrance would not diminish Ab-oligo staining of other antigens.

**Fig. 4:**
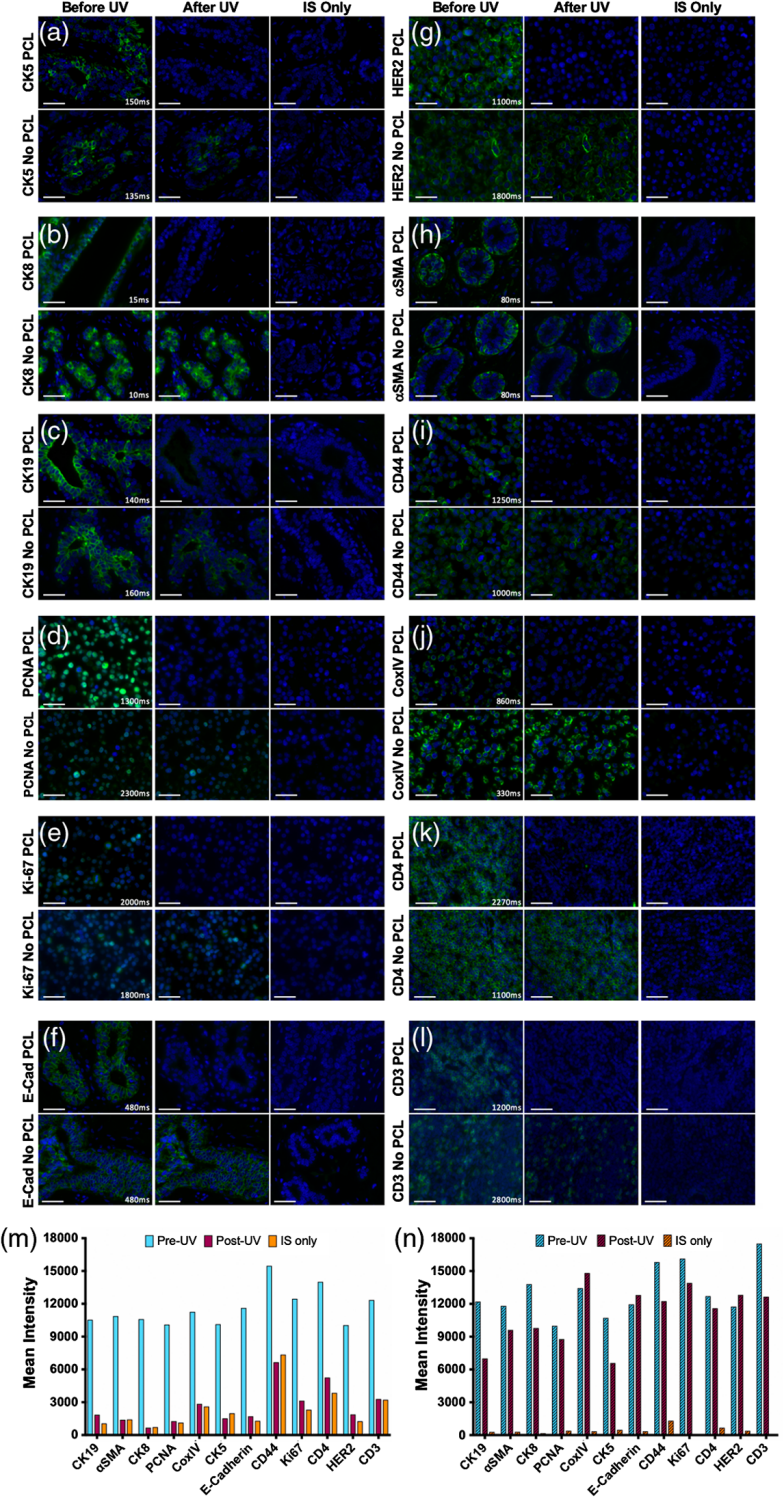
Ab-oligo signal removal validation using PCLs. Ab-oligo conjugate signal removal using PCLs was validated by staining serial sections using Ab-oligo conjugate (1°) and IS without a PCL as well as Ab-oligo conjugate and IS with a PCL. Following image collection to demonstrate the Ab-oligo tissue staining pattern, each sample was treated for 15 min with UV light and images were again collected of each sample. Serial sections were stained with IS only and imaged as a negative control. The Ab-oligo staining pattern before and after UV treatment was verified for (a) CK5, (b) CK8, (c) CK19, (d) PCNA, (e) Ki67, (f) E-Cad, (g) HER2, (h) α-SMA, (i) CD44, (j) CoxIV, (k) CD4, and (l) CD3. The mean fluorescence intensity for each image before and after UV light treatment was quantified for samples stained (m) with and (n) without a PCL. The 40-μm scale bars are displayed in all images.

Serial sections of the same test tissue per antigen (Table 1) were stained with IS containing fluorophore on both the $3^{\prime }$ and $5^{\prime }$ ends without PCL and with PCL (Fig. 4). Images of the staining pattern collected prior to UV light treatment demonstrated variable staining intensity with the addition of the PCL to the two fluorophore IS, with some targets showing similar mean fluorescence intensity with and without the PCL [Figs. 4(a), 4(d), 4(f), and 4(j)], some showing lower mean fluorescence intensity with the PCL [Figs. 4(b), 4(c), 4(e), 4(h), 4(i), 4(k), and 4(m)], and one showing improved mean fluorescence intensity with the PCL [Fig. 4(l)]. Negative control slides stained with the IS only, where either the IS with or without the PCL was used, showed that no appreciable signal from nonspecific staining or tissue autofluorescence contributed to overall staining intensity (Fig. 4). Antibody-specific fluorescence signal was completely removed in the PCL containing IS stained samples, where signal intensity was reduced to levels akin to IS only negative control samples. As expected, the Ab-oligo staining pattern was maintained when the IS without PCL was used for staining, although some signal was lost after UV light treatment, which was likely due to photobleaching of the fluorophore label with the high energy UV light (Fig. 4).

### 3.4 Multicolor Cyclic Immunofluorescence Staining and Visualization

**Fig. 5:**
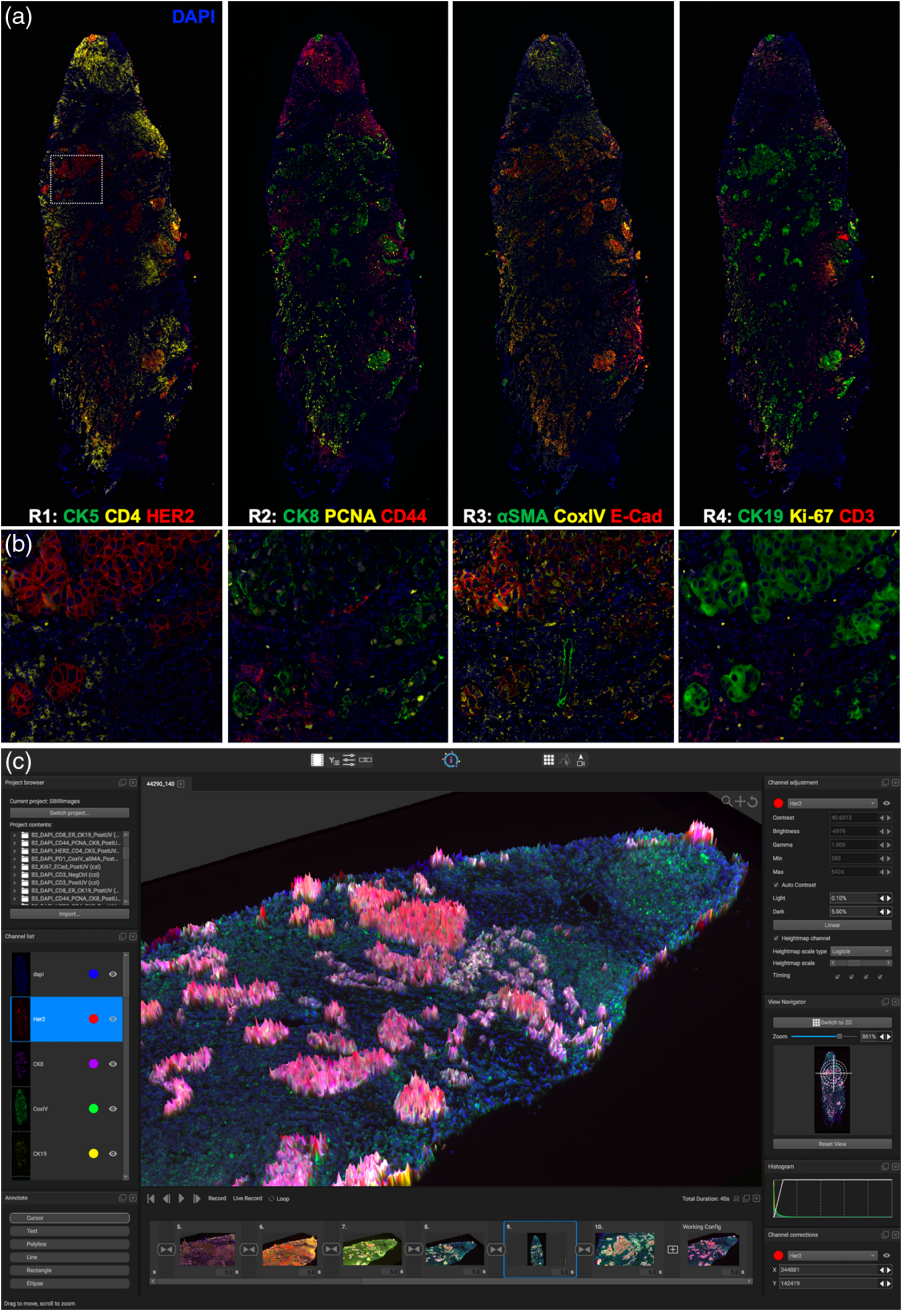
Multiplexed Ab-oligo cyCIF on HER2+ BC. (a) Tiled and stitched, whole breast cancer tissue images of Ab-oligo cyCIF are displayed by round of IS application. (b) A magnified view of a breast cancer tumor nest within the tissue sample (white box) is displayed for a higher resolution view of the staining pattern of each marker in their respective round of imaging. (c) QiTissue software was used to generate enhanced visualizations of this Ab-oligo cyCIF imaging data set by displaying CK8 as a height map. The height map is colorless, resulting in enhanced visualization of colocalization of markers without increased false coloring.

**Fig. 6:**
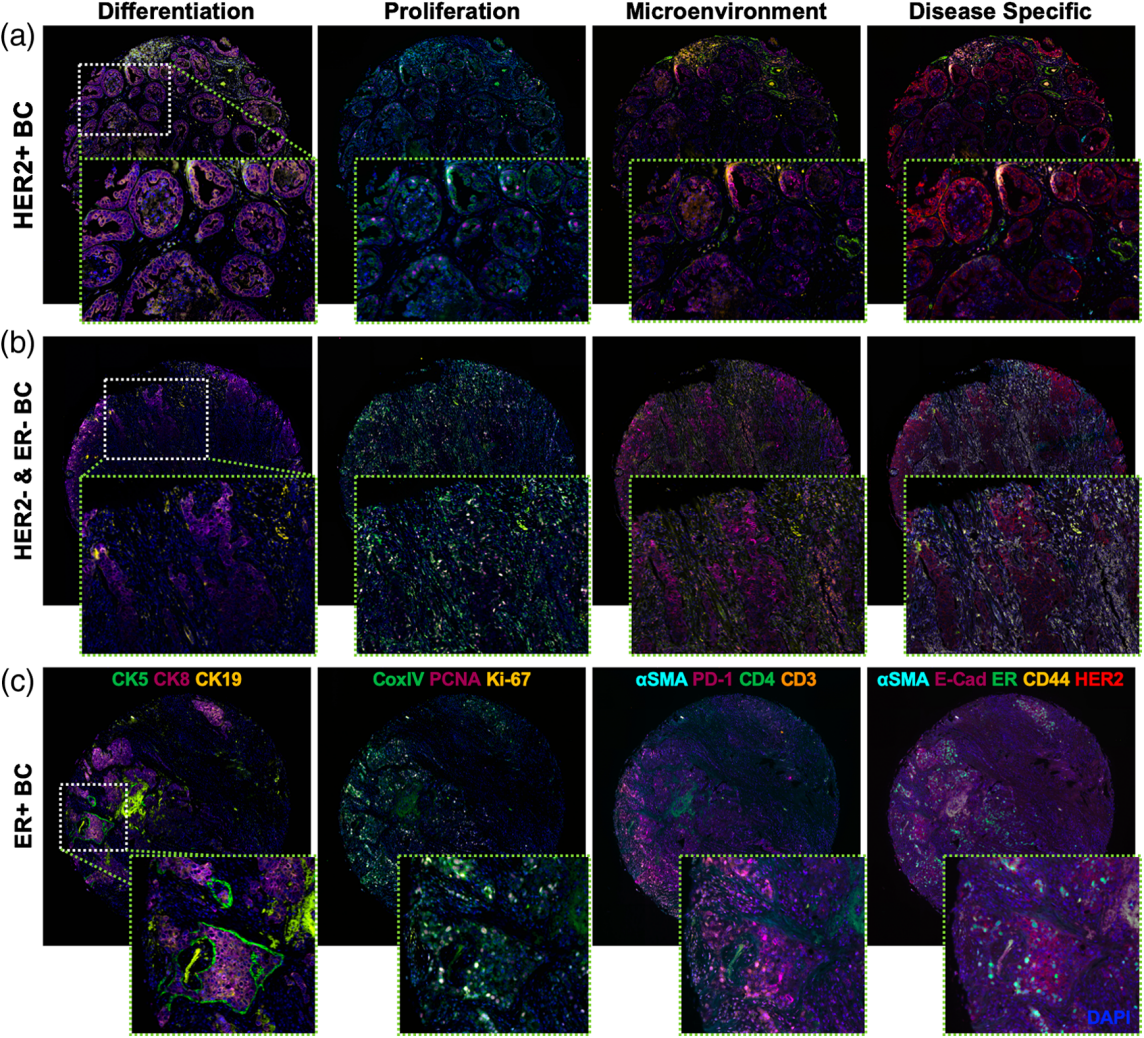
Fourteen-color Ab-oligo cyCIF on BC subtypes stained in a BC TMA. The validated Ab-oligo conjugates were used to stain a TMA containing different subtypes of BC. The biomarkers in the collected images were organized into tissue characterization panels of differentiation (CK5, CK8, and CK19), proliferation (CoxIV, PCNA, and Ki67), microenvironmental (α-SMA, PD-1, CD4, and CD3), and disease-specific (α-SMA, E-Cad, ER, CD44, and HER2) biomarkers, which were then used to characterize (a) HER2+, (b) HER2− and ER−, and (c) ER+BCs.

The validated set of Ab-oligo conjugates and PCL containing IS were used to generate multiplexed IF images of both a HER2+ breast cancer resection specimen (Fig. 5) and a breast cancer TMA (Fig. 6). The cocktail of Ab-oligo conjugates was applied as a single stain, while IS was applied in groups of three, where each IS group had spectrally distinct fluorophore labeling. DAPI staining was used in each imaging round for registration and to monitor for any potential tissue loss throughout the cyclic staining experiment. Tissue scanning was performed in each IS staining round, permitting image tiling for visualization of the entire specimen [Fig. 5(a)]. A magnified field-of-view demonstrated the spatial resolution of the Ab-oligo cyCIF technique, where individual cell staining patterns were readily visualized [Fig. 5(b)]. Staining of the HER2+ breast cancer tissue by round of IS staining showed variation in biomarker expression across the resection specimen for all interrogated biomarkers. As expected, the breast cancer epithelial cell marker HER2 as well as CK8 and CK19 was highly expressed in the breast cancer tumor nests. Proliferative cells, marked by Ki67 and PCNA, were also largely localized to the breast cancer tumor nests. Immune markers, including CD3 and CD4, were localized to the periphery of the tissue sample, mostly isolated from the breast cancer epithelial cells [Figs. 5(a) and 5(b)]. Even with only 12 biomarkers imaged, the difficulty with simultaneous visualization becomes apparent as overlays of more than four to five colors in any single, static image were challenging to visualize and interpret. Using the QiTissue Software, static visualization was enhanced using additional visualization tools, such as height maps to aid in data display [Fig. 5(c)].

Multicolor cyCIF staining in a breast cancer TMA was completed using the same rounds of three color IS application and UV light treatment. However, to help demonstrate the power of cyCIF, the biomarkers were remixed into biologically relevant groups, including differentiation (CK5, CK8, and CK19), proliferation (CoxIV, PCNA, and Ki-67), microenvironmental (α-SMA, PD-1, CD4, and CD3), and breast cancer-specific (α-SMA, E-Cad, ER, CD44, and HER2) markers rather than by imaging round (Fig. 6). Visualization of the selected biomarkers on different breast cancer subtypes showed the expected distinct expression patterns for HER2+ [Fig. 6(a)], HER2−, ER− [Fig. 6(b)], and ER+ [Fig. 6(c)] breast cancers. As expected, the HER2+ breast cancer had the highest HER2 staining intensity, while the ER+ breast cancer had the highest ER staining intensity. CK5 was only expressed in the ER+ breast cancer, with minimal expression in either of the other two breast cancer subtypes. CK8 and CoxIV showed ubiquitous expression across the three breast cancer subtypes, while α-SMA was found mainly in benign ductal breast tissues (Fig. 6). Tissue loss per round was quantified for all three TMA cores displayed by automated counting of DAPI labeled nuclei in each round of imaging, resulting in an average tissue loss per round of 0.52%±0.37.

## 4 Discussion

One of the primary goals of highly multiplexed immunolabeling and imaging of cancers is to unravel the complex biology and spatial organization that give rise to heterogeneity of therapeutic response. Although there have been a number of high-dimensional imaging techniques developed with the potential to interpret tumor heterogeneity, none have been adopted into the clinical histology laboratory, limiting their utility to aid in clinical decisions. Strategies using sequential rounds of biomarker staining, imaging, and destaining or antibody removal offer a potential near-term solution to build high-dimensional imaging data. However, tissue damage caused by sequential rounds of labeling and signal removal along with prohibitively long processing times to adopt said strategies has prevented widespread clinical use.

The validation studies of the Ab-oligo cyCIF technology presented herein highlight its ability to overcome the limitations of conventional immunolabeling technologies and avoid the pitfalls of alternative high-dimensional cyclic staining platforms. The employment of a site-directed conjugation strategy for the covalent conjugation of unique oligonucleotide sequences (i.e., DS), to the carbohydrate domains on the Fc region of the antibody resulted in maintained antigen binding affinity for the majority of antibody clones used to generate the Ab-oligo conjugates. Optimization of the SBR of the Ab-oligos for detection of antigens was completed first with a titration study of the Ab-oligo conjugate to determine optimal antibody concentration [Figs. 1(a) and 1(b)]. While a rather high optimal antibody conjugate concentration was selected herein (i.e., 15  μg/ml) to generate maximum SBR, all tested concentrations produced positive staining patterns, providing evidence that antibody staining concentrations and subsequent costs can likely be reduced. Additionally, further Ab-oligo titration is ongoing to assess optimal concentrations for other antigens. SBR optimization was also maximized through IS titration studies (Fig. 1) using an antigen localized to each cellular compartment: membrane (E-Cad), cytoplasm (α-SMA), and nucleus (Ki67). This was by design to understand whether antigen location affected DS/IS hybridization and whether optimal IS concentration varied with cellular compartment. An IS concentration of 350 nM was found to generate the highest SBR across markers with little difference in intracellular localization noted [Figs. 1(c) and 1(d)]. Subsequent qualitative Ab-oligo staining pattern validation studies using a 15  μg/ml Ab-oligo conjugate and 350 nM IS as the optimal staining methodology for all markers showed the expected target-specific staining patterns for the Ab-oligo cyCIF strategy, when compared with matched indirect IF-positive controls (Fig 2). Ab-oligo cyCIF sensitivity was found to be similar to conventional direct IF using antibodies with similar fluorophore to antibody CRs [Table 1 and Fig. 2(g)].

Successful cyclic immunostaining requires robust fluorescent antigen labeling with subsequent full signal removal between staining rounds. Improved Ab-oligo fluorescent SBR was generated through the addition of a second fluorophore to the IS configuration, enhancing SBR for both HER2 and E-Cad [Figs. 3(a), 3(b), and 3(d)]. The IS length was subsequently evaluated to ensure steric hindrance with a fluorophore on the $3^{\prime }$ end, which would require hybridization next to the antibody, was avoided. An optimal IS length of 26 nt with a fluorophore and an adjacent PCL on each end was selected for all future studies [Figs. 3(c) and 3(e)]. The selection of the shortest tested length mitigated the chance for steric hindrance in high-dimensional staining studies in our sequential imaging strategy.

Antibody stripping and fluorophore bleaching techniques are commonplace in cyclic immunostaining; however, their use is often to the detriment of tissue integrity. Herein, we sought a mild signal removal technique that would preserve tissue integrity over multiple staining cycles. We found the incorporation of PCLs into the IS configuration to provide complete signal removal after exposure to UV light [Figs. 4(a)–4(m)]. Differences in overall signal intensity before and after UV treatment varied depending on the stained antigen [Fig. 4(g)]; however, no recognizable staining pattern remained after UV signal removal, validating the utility of PCLs for cyCIF [Fig. 4(n)]. The main reason for the differences in signal intensity following UV treatment was variation in background autofluorescence signal present in the different FFPE sample types used for validation.

Key benefits to Ab-oligo cyCIF are (1) the use of oligo labeled antibodies permits a single long antibody staining step as all Ab-oligos can be added simultaneously, (2) PCLs for signal removal do not damage tissue integrity, and (3) the strategy results in a simple workflow using only conventional reagents and fluorophores for ready translation to any laboratory or clinical setting. Validation and optimization of our Ab-oligo cyCIF platform enabled the extension of our technique to generate up to 14-color images on multiple HER2+ and/or ER+ BC tissue as conventional FFPE blocks (Fig. 5) or TMAs (Fig. 6). The high-dimensional immunolabeling and imaging on these samples demonstrated the utility of cyCIF to visualize complex tumor heterogeneity *in situ*. Complete signal removal between sequential rounds of staining and imaging was achieved to avoid cross talk between multiple markers labeled using the same fluorophore. Successful photocleavage and simple washing of UV light treated samples prevented accumulation of background autofluorescence as the number of staining rounds increased. Importantly, the process of signal removal by UV light treatment, multiple applications of IS, and multiple rounds of imaging did not cause any appreciable tissue damage. This observation gives motivation to further extend Ab-oligo cyCIF to higher dimensionality in future work. The simple workflow using a single antibody incubation step and sequential rounds of target imaging was validated herein. Additionally, it is important to note that all antibodies applied prior to the first round of IS application were still present in the fourth round of imaging, resulting in antibody-specific fluorescent staining patterns when labeled with their complementary IS. This validated simple workflow with mild staining and destaining conditions presents the opportunity for automated staining and imaging using emerging fluorescence microscopy technologies with robotic staining solutions.

Our validated Ab-oligo cyCIF platform employs a workflow capable of producing high dimensionality imaging from a single tissue sample without deleterious effects to tissue or requiring exotic reagents or microscopy hardware, decreasing the barrier to entry for cyCIF into the clinical setting. While biological interpretation is beyond the scope of this paper, tools for analysis and interpretation of high-dimensional image data sets produced from Ab-oligo cyCIF will be critical for clinical decision making. Herein, we utilize QiTissue software for visualization; however, additional tools (e.g., histoCAT^29^^,^^30^ and PhenoGraph^31^) have been developed to perform such analyses on datasets produced by alternative multiplexed imaging technologies and are compatible for analysis of Ab-oligo cyCIF data. These analysis tools use the high-dimensional data to identify the unique cell phenotypes and assess the significance of each cell subpopulation in a tumor. The single cell resolution of these analyses simultaneously informs on cell-to-cell interactions and the complex interactions between cells and their tumor microenvironments to draw clinically impactful conclusions. Important to clinical potential, Ab-oligo cyCIF results in biomarker fluorescent labeling SBR equivalent to conventional direct IF methods, validating it as a sensitive quantitative assay for detection of clinically relevant proteins (e.g., HER2) with intertumoral expression variation. The addition of a dual fluorophore IS configuration was critical to this result, and additional signal amplification studies are ongoing to further improve Ab-oligo cyCIF SBR to reach the levels of indirect IF. Furthermore, additional Ab-oligo conjugates are currently under development to address therapeutic response heterogeneity in breast, pancreatic, and nonsmall cell lung cancers. One such application will be to quantify cellular pathway reprogramming as a mechanism of acquired resistance in response to targeted therapies. Ab-oligo targets will be extended to include total protein and phosphorylated protein targets to quantify protein signatures critical to pathways that are targets of therapeutic inhibition, where Ab-oligo cyCIF could aid in the identification of novel therapeutic strategies for a wide range of disease states.
